# Aluminium Matrix Composites Reinforced with AlCrFeMnNi HEA Particulates: Microstructure, Mechanical and Corrosion Properties

**DOI:** 10.3390/ma16155491

**Published:** 2023-08-06

**Authors:** Elias A. Ananiadis, Alexandros E. Karantzalis, Athanasios K. Sfikas, Emmanuel Georgatis, Theodore E. Matikas

**Affiliations:** 1 Department of Materials Science and Engineering, University of Ioannina, 45110 Ioannina, Greece; i.ananiadis@uoi.gr (E.A.A.); mgeorgat@uoi.gr (E.G.); 2 Faculty of Engineering and Environment, Northumbria University, Newcastle upon Tyne NE1 8ST, UK; athanasios.sfikas@northumbria.ac.uk

**Keywords:** aluminium matrix composites, particulate reinforcement, AlCrFeMnNi high entropy alloy reinforcement, nanoindentation, creep, sliding wear, corrosion, potentiodynamic polarisation

## Abstract

Novel aluminium matrix composites have been fabricated using a powder metallurgy route with reinforcement phase particles of high entropy alloy (HEA) consisting of third transition metals. These new composites are studied as far as their microstructure (SEM, XRD), basic mechanical properties (hardness, elastic modulus) and creep response using nanoindentation techniques are concerned. Wear (sliding wear tests) and corrosion behaviour (in 3.5 wt.% NaCl environment) were also assessed. It was observed that, microstructurally, no secondary intermetallic phases were formed. Hardness and wear resistance seemed to increase with the increase in HEA particles, and in terms of corrosion, the composites exhibited susceptibility to localised forms. Nanoindentation techniques and creep response showed findings that are connected with the deformation nature of both the Al matrix and the HEA reinforcing phase.

## 1. Introduction

Metal matrix composites (MMCs) involve two or more phases, where the dominant phase is the matrix and the other phase can be different types of reinforcement. Reinforcement may be either metallic, non-metallic or ceramic, used to obtain/achieve enhanced properties, physical and mechanical. This addition improves tribological properties and enhances specific strength and hardness. MMCs find wide applications in different sectors such as aerospace, transportation and others [1,2,3,4,5].

A new approach to reinforcement of these composites is the addition of high entropy alloy (HEA) particulates. HEAs are alloys that contain at least five principal metals with concentrations from 5 to 35 at.%. These novel types of metallic materials present exceptional properties (physical, mechanical, corrosive, etc.) that are derived from four core elements: (i) the high entropy effect, (ii) the cocktail effect, (iii) severe lattice distortion and (iv) sluggish diffusion [6,7].

These new alloys are a good choice for reinforcement phase in aluminium matrix composites (AMCs) due to the sluggish diffusion behaviour, their outstanding properties and less reactive nature [8,9]. AMCs have been fabricated through a variety of techniques including ultrasonic casting [10], hot pressing [11], selective laser melting [12], stir casting [13,14,15], spark plasma sintering [16,17] and microwave sintering [18]. Different types of HEA reinforcements have been studied including AlxCoCrFeNi [10,12,13], CoNiFeCrAl0.6Ti0.4 [11], CoCrFeMnNi [14,15], CoMoMnNiV [16] and Fe1.2CrZnCuAlTi0.8 [17]. AMCs reinforced by HEA particles exhibited higher hardness and better mechanical properties as compared to the monolithic matrix material [10,11].

Hardness, elastic moduli and creep examination in nanoscale can be obtained from the nanoindentation technique. In metal composites, conventional techniques are not capable to measure the mechanical properties of the interface between the particles and the matrix due to the micro-based dimensional scale. Nanoindentation can assess the mechanical behavior at these specific regions because it can measure the plastic deformation in such small volumes [19,20]. Creep resistance may be one of the most significant properties for industrial applications. For the creep evaluation, macroscale-based tensile methods are used, but in terms of microscale, nanoindentation methods were applied successfully. The first creep evaluation with nanoindentation was analysed by Li et al. [21] in different materials.

Tribological behaviour is one of the most researched responses of the MMCs. Many different key parameters such as applied load, sliding distance, sliding speed, reinforcement particles, matrix etc., have been reviewed by different researchers. Different wear mechanisms have been proposed either individually or in combination in order to evaluate the involved wear mechanisms. All these years of extensive study have led to the conclusion that the sliding wear response of AMCs is regulated by a transition between three modes, namely “light”, “mild”, and “severe” [22,23,24]. The most common mechanisms involve (i) the delamination theory of Suh where material loss is caused by crack/flaw formation and propagation as a result of plastic deformation [25], (ii) the basic Archard hypothesis, according to which material removal involves an extensive and repetitive adhesion–decohesion process of the two counter bodies and (iii) the tribolayer approach, in which surface-formed oxide phases are mechanically mixed with the metallic matrix to form a dynamic surface layer that is continuously subjected to a comminution–consolidation action, the stability of which regulates the severity of the degradation phenomena [23,26,27].

Corrosion performance of Al metal matrix composites has been widely studied. In more detail, the effects of different types of particle reinforcement have been assessed, including SiC [28], WC [29], TiC [29] and intermetallic compounds [30]. The introduction of the reinforcement may have a beneficial [28], neutral [29] or negative [31] role in the corrosion performance of Al matrix composites. Very few works have focused on the corrosion resistance of Al matrix composites reinforced by HEA particulates. Han et al. studied the corrosion performance of Al6061 reinforced by CoCrFeNi particles (15 vol.%) fabricated by cold spray and subsequent friction stir processing. According to their results, the corrosion behaviour of the composites appears to be controlled by the corrosion of the matrix phase [32]. Wang et al. focused on assessing the corrosion performance of Al reinforced by different volumes of CuZrAlTiNiW via mechanical alloying and subsequent spark plasma sintering. The introduction of the HEA particles enhances the pitting resistance of the material in seawater, with the composite containing 30 vol.% exhibiting the best corrosion performance [33]. In a previous work of the authors [34], the corrosion resistance of Al matrix composites reinforced by refractory HEA particulates (1–5 vol.%) was assessed. According to their findings, the introduction of reinforcing particles does not appear to have a significant effect on the corrosion resistance of the composite.

The aim of this study is to evaluate the feasibility of producing AMC reinforced by different volume fractions of HEA particulates with the employment of a powder metallurgy fabrication route. Additionally, this work aims to study the effect of different particulate volumes on the microstructure and properties. A variety of material properties have been assessed, including mechanical properties, sliding wear response and corrosion resistance. More specifically, the present effort focuses on the use of a new HEA type of reinforcement which, to the best of the authors’ knowledge, has not been tested before and to evaluate specifically the comparative effect the increase in the reinforcing phase has on a series of properties. 

## 2. Materials and Methods

### 2.1. Fabrication

The reinforcement material, high entropy alloy AlCrFeMnNi, was produced via vacuum arc melting technique. Each specimen was melted and followed by another 5 remeltings to ensure homogeneity of the material. Then, the HEA was crushed into powder by dry grinding using a planetary mill (Fritsch Pulverisette 7 Premium Line, Markt Einersheim, Germany). The samples were put into a WC bowl with capacity of 80 mL along with 10 WC balls of 15 mm in diameter. Τhe process took place in intervals, working 10 min and pausing 20 min, repeated for 4 times. Intervals were used to prevent overheating the material and its sticking on the bowl and balls’ surface. Detailed analysis of the final powder form is found in the results.

Pure aluminum AA-1050 (99.5%) was used as matrix material and the HEA as the reinforcement phase to produce composites in three different compositions (1%, 3% and 5% vol.).

The subjected samples of this research were fabricated via a powder metallurgy route. Powders of aluminum and HEA were weighted in the chosen composition and stirred with methanol into a beaker to avoid agglomeration and ensure decent dispersion, letting them dry on a hot plate. The powders then were pressed into cylindrical pellets of 14 mm in diameter with a hydraulic press. The cold press samples were carried into steel dies with 16 mm diameter (slightly bigger than the samples to avoid breaking the sample during insertion) covered with graphite powder for lubrication and sintered in a hot press furnace (manufactured by Termolab, Agueda, Portugal). The sintering stage was carried out in the following steps: First, the temperature was raised to 550 °C with a step of 50 °C/min with inert atmosphere using argon gas. The temperature was held at that point for 1 h and then the second step was initiated, which included the loading process. The applied load was 6500 N. It was gradually raised at a rate of 100 N/min and held for 30 min. Finally, the composites were allowed to cool into the furnace.

### 2.2. Scanning Electron Microscopy

A Scanning Electron Microscope (JEOL 6510 LV, Tokyo, Japan) was used for the study of the microstructure of the composites, the surface evaluation after the corrosion experiments and the aftermath of the wear assessment on the surface wear tracks and wear debris.

These areas of interest were also examined by EDX (energy dispersive X-ray spectroscopy) analysis (X-Act EDS by Oxford Instruments, Abingdon, UK).

### 2.3. X-ray Diffraction and Hardness Testing

For X-ray diffractograms, D8 ADVANCE from Bruker was used (Billerica, MA, USA), and for the hardness measurements, an Innovatest IN-700M universal hardness tester was facilitated. A Brinell 5 scale was chosen, and at least five measurements were taken for each specimen.

### 2.4. Nanoindentation and Creep Testing

For the nanoindentation and creep evaluation, a Shimadzu DUH-211S nanoindenter (Kyoto, Japan) was used. The depth for each experiment was set at 1000 nm, the maximum applied load of the instrument was set at 196.13 mN and two different loading speeds were selected, a “fast” one at 13.3240 mN/s and a “slower” at 2.2207 mN/s. These conditions were applied for nanoindentation measurements and creep, but in this later case, the applied load was held for 30 s. The areas of interest of the conducted experiments were the bulk HEA reinforcement and the matrix of the 5 vol.% Al-HEA composites. Data from each measurement were acquired and provided by Shimdazu’s software (Version 2.40).

### 2.5. Wear Testing

Wear experiments were conducted on a ball-on-disc set up (CSM Instruments tribometer, CSM Instruments, Needham, MA, USA) with a steel ball (100Cr6) used as a counter body. The speed was 10 m/s, and the sliding distance was set at 1000 m. Every 200 m, the wear test was interrupted for the specimen to be weighted. Finally, the mass loss and the wear rate were calculated.

### 2.6. Corrosion Testing

Corrosion study was carried out by cyclic polarisation technique in a Gamry Reference 600 potentiostat/galvanostat with a standard three electrode cell. The specimen was set as the working electrode, a graphite plate was used as the counter electrode, and as reference electrode, a saturated calomel (SCE) one was selected. The corrosion experiments were conducted into 3.5 wt.% NaCl solution (simulating sea water). A buffer solution was mixed with the water to set the pH at 7. After 1 h of immersion of the samples in the solution, the open circuit potential was determined. Finally, the scan rate of the polarisation was 10 mV/min.

## 3. Results and Discussion

### 3.1. Microstructural Analysis

**Figure 1:**
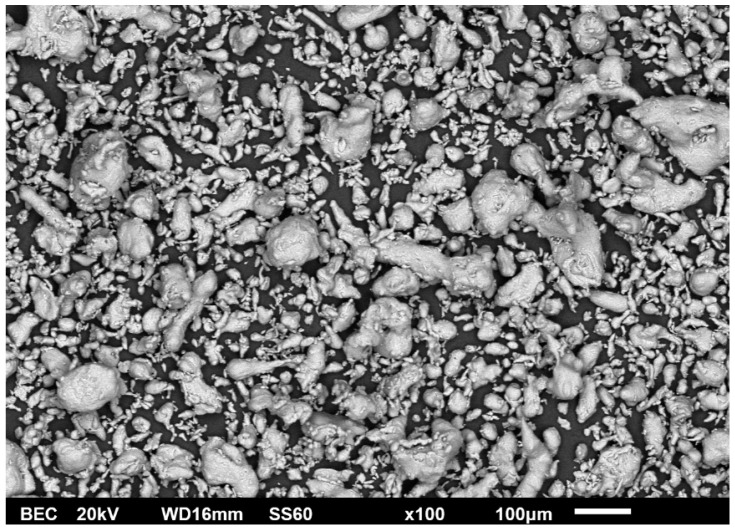
SEM image for aluminium atomised powder.

The aluminium powder that was used as the composites’ matrix phase is presented in Figure 1. It can be observed that, despite the fact that the powder was produced by atomisation, its shape is of an elliptical/ligament-like morphology rather than spherical. What is also important to mention is that the size of the particles covers a wide range from 1–10 μm up to 100–150 μm.

**Figure 2:**
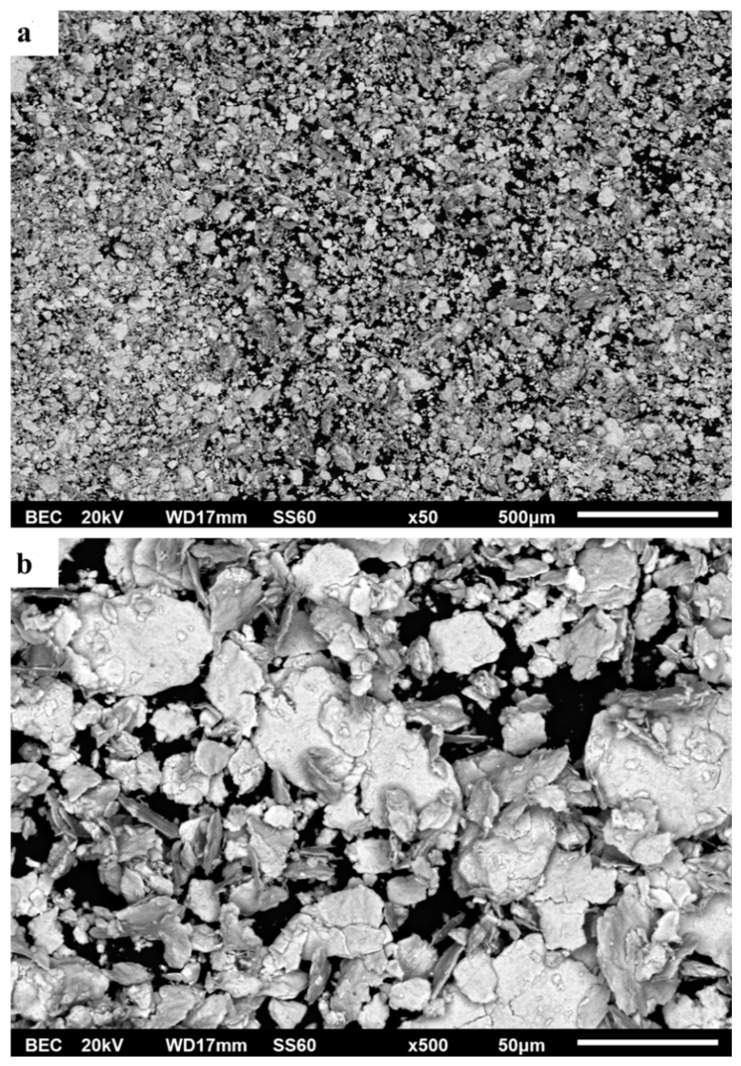
Images from the milled powder of AlCrFeMnNi HEA in (**a**) panoramic view (×50) and (**b**) higher magnification (×500).

Figure 2a,b shows the morphology and shape of the reinforcing HEA particles after the milling process. It can be observed that different morphologies and sizes are present. The fine-size particles are of an equi-axed shape, whereas the coarse particles possess more flake-like and angular morphologies. Using Image J image processing software, it was found that the size of the fine particles ranges between 1–10 μm and the coarse particles lay within a range of 50–100 μm.

**Figure 3:**
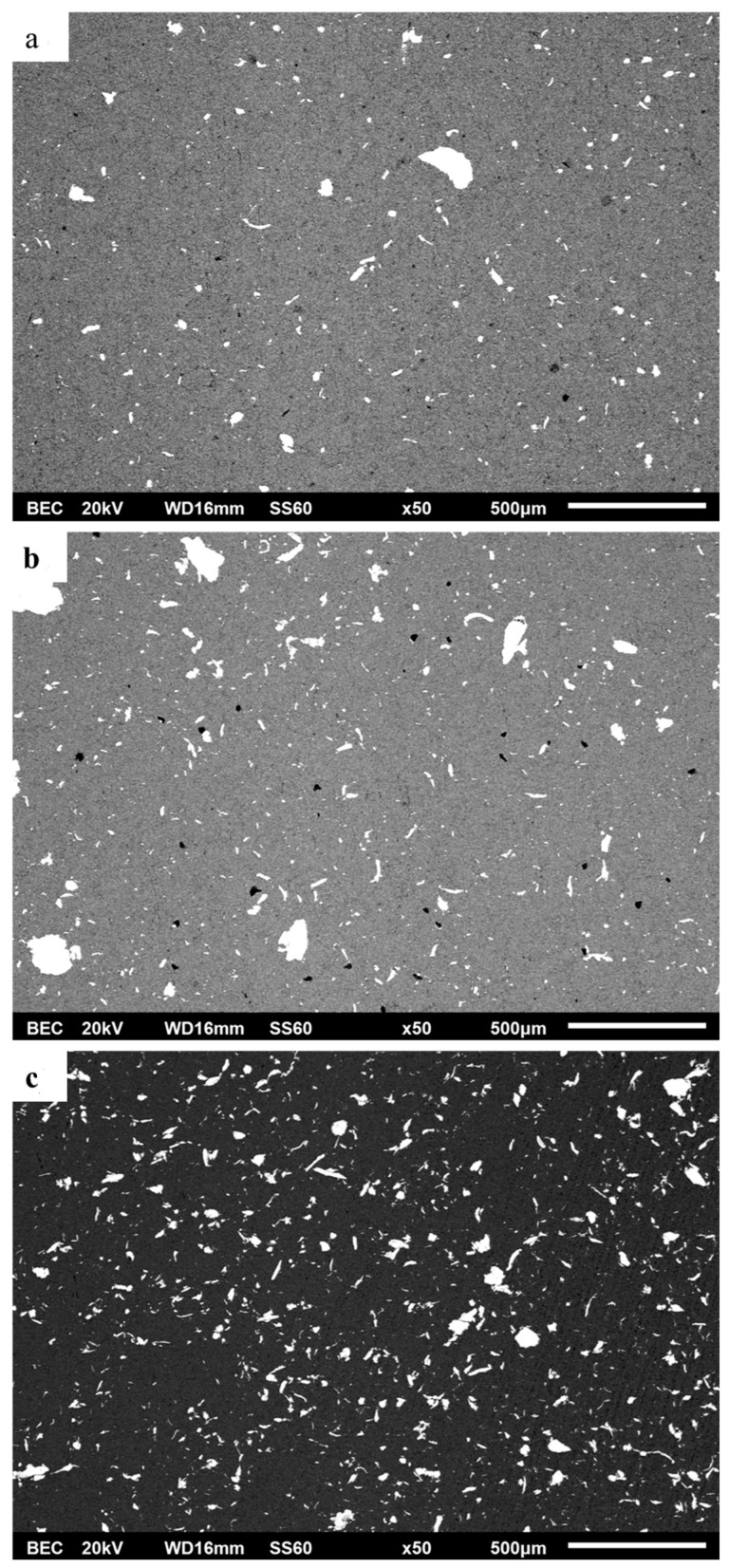
(**a**) Panoramic view of the microstructure of the 1 vol.% AlCrFeMnNi-reinforced composite. (**b**) Panoramic view of the microstructure of the 3 vol.% AlCrFeMnNi-reinforced composite. (**c**) Panoramic view of the microstructure of the 5 vol.% AlCrFeMnNi-reinforced composite. In all cases, the presence of black spots indicate the existence of some porosity.

Figure 3a–c shows a panoramic view of the microstructure of the different produced composites after SEM examination under back-scattered mode (BSC mode). Based on these microstructural images, the following points can be addressed.

The reinforcing particle content seems to be in agreement with the nominal/targeted compositions. Using ImageJ software, the percentage of area surface covered by the reinforcement was calculated at 2.04% for 1 vol.% composite, 4.31% for 3 vol.% composite and 7.18% for 5 vol.% composite.

Both coarse and fine particles are considerably homogeneously distributed within the matrix phase. As the reinforcement content increases, it appears the particle distribution becomes even more homogeneous (Figure 3c).

Some porosity, in the case of the 1 and 3 vol.% reinforced composites (0.269% and 0.676%, respectively), can be observed (black dots in Figure 3a,b). Despite the presence of this minimum porosity, no other imperfections, such as cracks and/or flaws, can be observed. The lack of such defects implies an improved quality of the sintering process.

**Figure 4:**
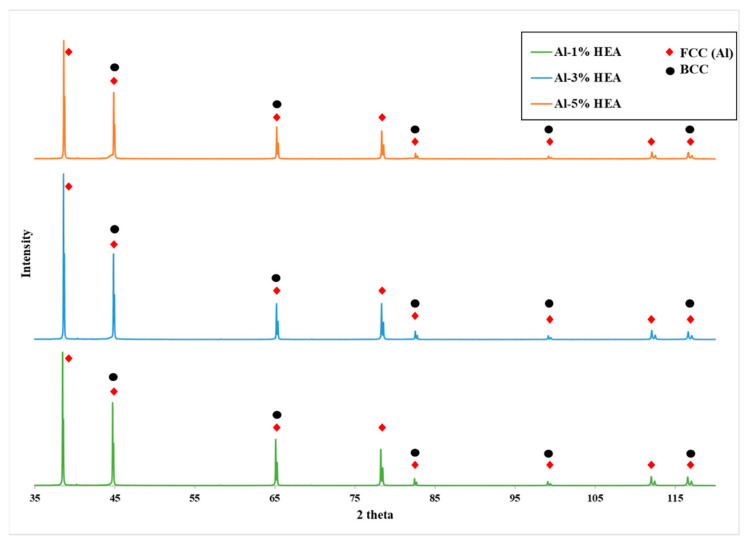
X-ray diffractograms of the produced composites.

In order to gain more information on the microstructural features of the produced systems, XRD analysis was performed, the results of which are presented in Figure 4. It can be observed that the Al matrix phase can be identified in all the different diffractograms, and the presence of the AlCrFeMnNi particle phase can be observed at some peaks (characteristic of BCC phase).

**Figure 5:**
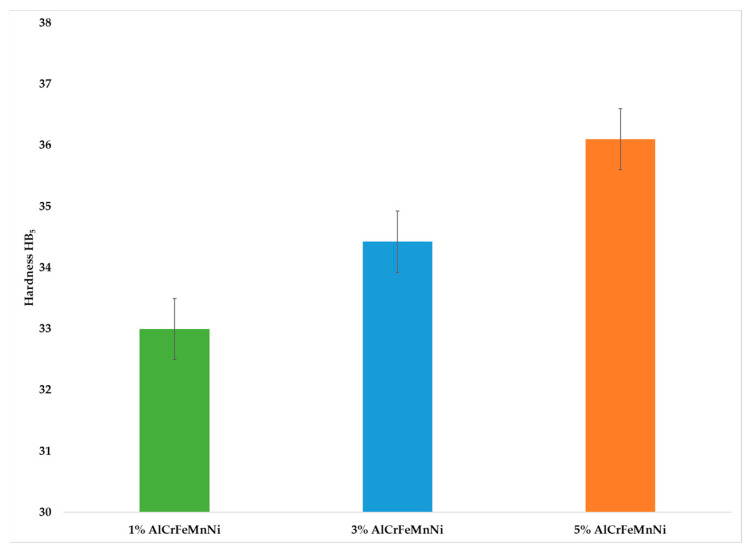
Hardness values for Al-HEA composites.

Hardness measurements for the three different composites are shown in Figure 5. The hardness increases as the reinforcement phase is increased. As the surface of the composites becomes denser with particles, the movement of mass tends to occur with more difficulty. 

**Figure 6:**
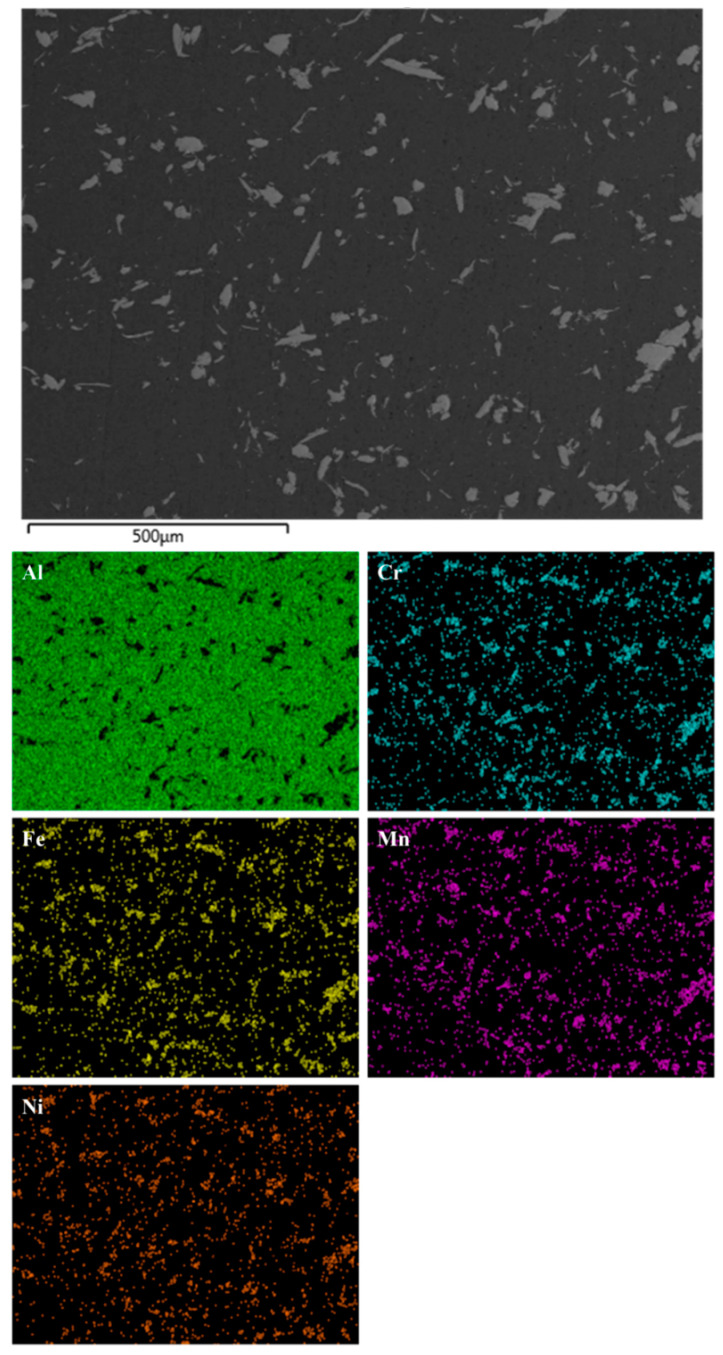
SEM-EDX chemical mapping in the case of the 5 vol.% composite. The elemental distribution and location verify that the reinforcing particles are of the HEA system.

**Figure 7:**
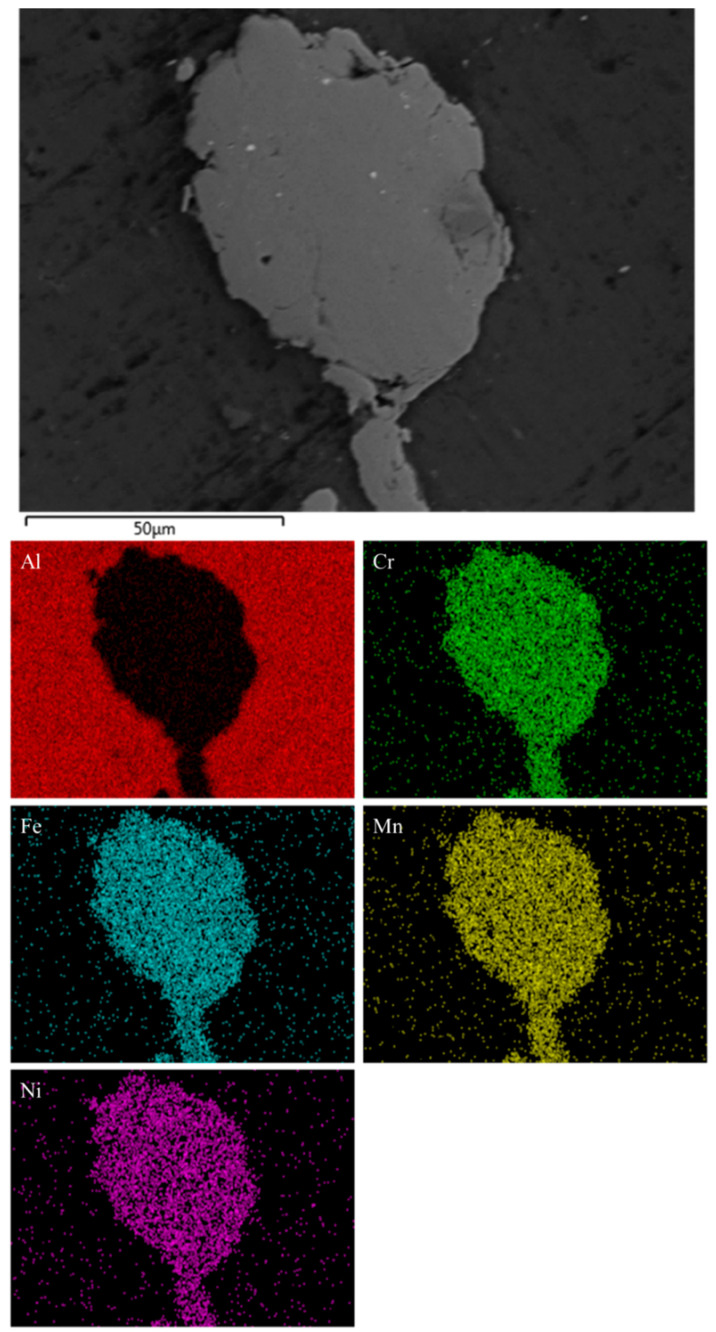
SEM-EDX chemical mapping of a coarse particle in the case of the 5 vol.% composite. The elemental distribution shows the HEA nature of the particle. Additionally, a rigid and continuous interfacial area seems to have been established.

The presence, however, of the HEA reinforcing particles has indeed been verified by SEM-EDX chemical mapping. Figure 6 shows the results of this analysis in the case of the 5 vol.% reinforced composite. Clearly, this elemental analysis shows that the reinforcing particles are of the AlCrMnFeNi high entropy alloy. This evidence becomes more profound in Figure 7, where the elemental mapping analysis of a large reinforcing particle is presented. Also worth noticing in Figure 7 is the quality of the particle–matrix interfacial area where no voids, no flaws and no undesirable reaction products, at least at this magnification, can be observed. To the contrary, a rigid and solid interfacial region seems to have been established.

### 3.2. Nanoindentation and Creep

#### 3.2.1. Basic Mechanical Property Assessment

**Figure 8:**
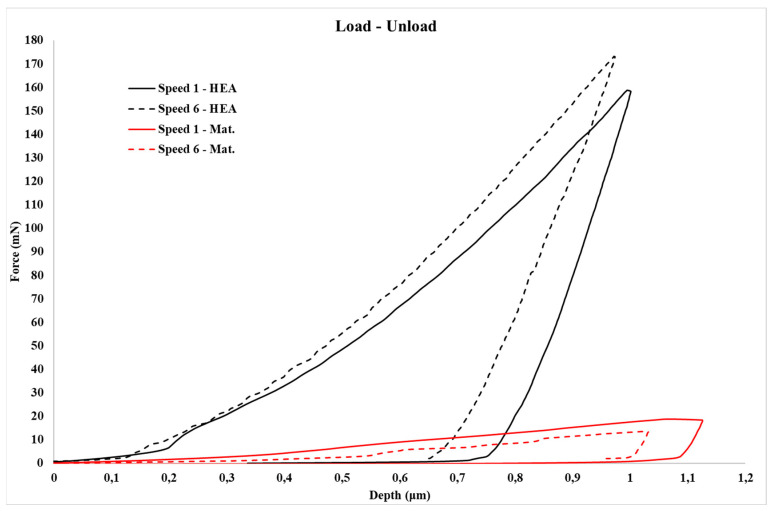
Nanoindentation load–unload cycle curves for matrix and HEA reinforcement.

Load–unload nano indentation curves are presented in Figure 8, at no creep stage. Basic mechanical properties (i.e., Young’s modulus, hardness, etc.) of nanoindentation are calculated based on these curves.

Table 1 and Table 2 (Speed 1 (13.3240 mN/s) and Speed 6 (2.2207 mN/s), respectively) present the values obtained from the measurements during the nanoindentation testing. As referred to previously, the basic mechanical properties acquired were the modulus of elasticity by nanoindentation, E_it_, the energy absorbed in the elastic region versus the overall absorbed energy, n_it_, and the hardness of the tested specimen in Vickers scale, HV.

According to the data from Table 1 and Table 2, the following findings are extracted:

- **Nanoindentation Hardness, HV**: Taking into consideration previous work [19], the hardness values follow a common trend. In more detail, the matrix exhibits considerably lower hardness values compared to those of the HEA particulates, which is expected as the AlCrFeMnNi HEA is a hard material of BCC and FCC phases with hardness around 57 HRC. Especially in the case of the matrix, as Table 1 and Table 2 show, at the condition of the lower loading speed, the hardness seems to exhibit a slight decrease. If we recall that hardness is practically the resistance to plastic deformation, it can be postulated that at lower loading speeds the plastic deformation is enhanced, as at lower speeds, both the time and ease for dislocation mobility are enhanced and simultaneously the probability for dislocation entanglement and related strain hardening phenomena are discouraged.
- **Modulus of Elasticity in Nanoindentation, *E_it_***: For the reinforcing phase, the values are very high. The specific HEA consists of BCC and FCC phases. Both phases establish strong interatomic bonds which in conjunction with the severe lattice distortion effect caused by the different atomic size in HEAs create strong elastic stress fields within the lattice, resulting in a considerably high modulus of elasticity [6,35]. The matrix phase on the other phase shows values that agree with the literature [36]. Even though Table 1 and Table 2 present a light increase at lower loading speed, the scatter of the obtained values most likely suggests that the loading speed does not clearly affect the values of the modulus of elasticity.
- **Ratio of the energy absorbed in the elastic region over the total absorbed energy, *n_it_***: The *n_it_* ratio follows the same tendency as the hardness (the resistance to plastic deformation as mentioned previously. As such, an increase in the hardness leads to an increase in the energy absorbed in the elastic region, meaning that the *n_it_* ratio will also increase.

#### 3.2.2. Creep Assessment

##### Calculations

For the appropriate calculations to be performed, the approaches used by Karantzalis et al. [37,38,39] and Zhang et al. [40] were adopted. A Berkovitch diamond indenter was used, and the parameters/values necessary for the calculations were the strain rate, the hardness as a function of the depth and the creep depth as a function of the creep holding time of indentation. The formulated equations are as follows:

(1) $$\dot{\varepsilon }=\frac{1}{h}\frac{dh}{dt}=\frac{\dot{h}}{h}$$

and

(2) $$H=\frac{P}{24.5h^{2}}$$

In these equations, the above ε refers to the strain rate, *h* is the depth of indentation as a function of time, *H* is the hardness and *P* is the applied load as a function of time.

Wang et al. [41] proposed a protocol that approaches the indentation displacement as a function of the holding time. This approach, with some minor modifications, can be used to obtain a fitting curve in the form of

(3) $$\Delta h\left(t\right)=h\left(t\right)-h_{0}=at^{p}+kt$$

With Δ*h*(*t*) being the net creep indentation depth, *h*(*t*) the depth of indentation as a function of the holding time, h_0_ the initial depth at the beginning of the creep stage, t the holding duration time during the creep stage and a, p and k the fitting parameters.

As in previous work of Karantzalis et al. [39], in the present work the creep stage initiates when a 1000 nm depth is established. At this point, the creep penetration depth is recorded as a function of time for a total duration of 30 s and under maximum load (*P_max_*, i.e., the load where the 1000 nm initial depth was reached). The pure depth due to creep can be derived by (4):

(4) $${h(t)}_{creep}=at^{p}+kt$$

and the overall indentation depth is

(5) $$h\left(total\right)=h_{0}+at^{p}+kt$$

where *h*_0_ is the indentation depth at the onset of the creep stage and is close to 1000 nm, which is the selected indentation depth.

According to Li et al. [21] and Yu et al. [42], creep in indentation follows an empirical law of the form:

(6) $$\dot{\varepsilon }=A\sigma^{n}\exp\left(-\frac{Q}{RT}\right)$$

Here *A* is a constant, *σ* the applied stress, *Q* the activation energy, *R* the gas constant and *n* is the creep stress exponent. Because all the tests are performed in ambient and room temperature conditions, the exponential factor of the previous equation can be assumed as constant. Therefore, Equation (6) can be reformed as

(7) $$\dot{\varepsilon }=\lambda \sigma^{n}$$

Βy applying the logarithmic function in both terms in Equation (7) we get

(8) $$\ln\left(\dot{\varepsilon }\right)=\ln\left(\lambda \right)+nln(\sigma )$$

Additionally, similar treatment in Equation (1) results in

(9) $$\ln\left(\dot{\varepsilon }\right)=ln(\frac{1}{h}\frac{dh}{dt})$$

Zhang et al. [40] proposed another equation which is very useful to conclude the overall approach:

(10) σ=kH

with k being a parameter related to the tested specimen.

Following this equation sequence, the stress exponent n can be calculated by plotting $\ln\left(\dot{\varepsilon }\right)$, i.e., $ln(\frac{1}{h}\frac{dh}{dt})$ with ln⁡(H). Once the stress exponent is calculated, another important parameter, the strain rate sensitivity, m, can also be calculated by

(11) $$m=\frac{1}{n}$$

The standard mechanical properties measured in the present effort (modulus of elasticity, hardness and nit) were calculated by the adopting the approach of Oliver and Pharr [43].

**Figure 9:**
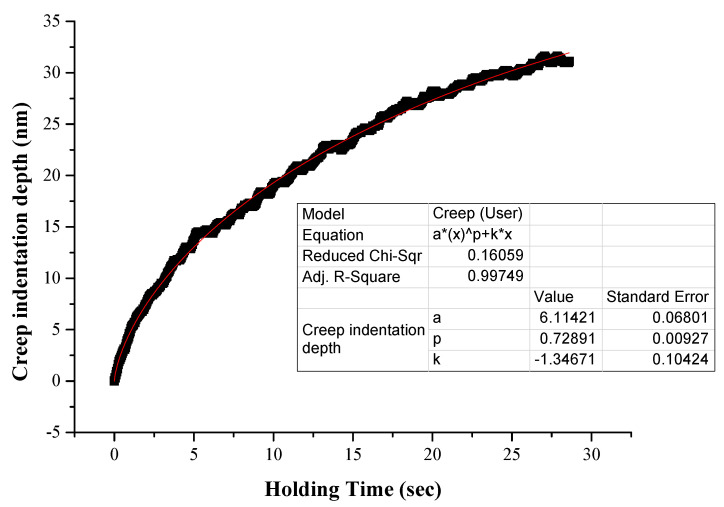
Power law fitting of actual creep depth versus time curve.

**Figure 10:**
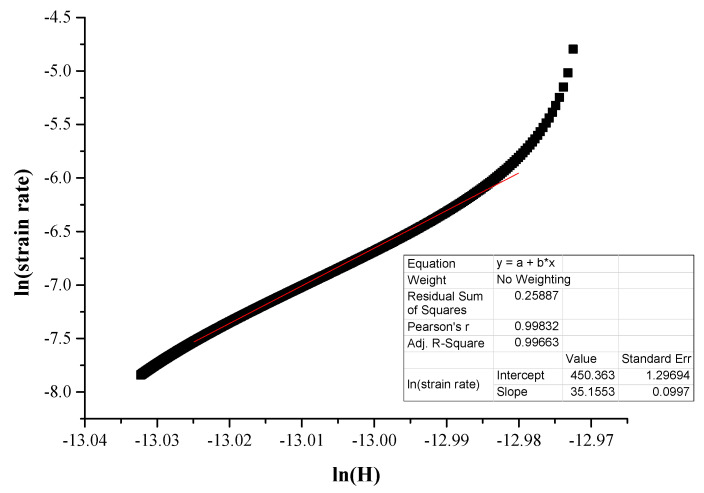
Linear fitting of steady creep area for extrapolated stress exponent (*n*) slope calculation.

**Figure 11:**
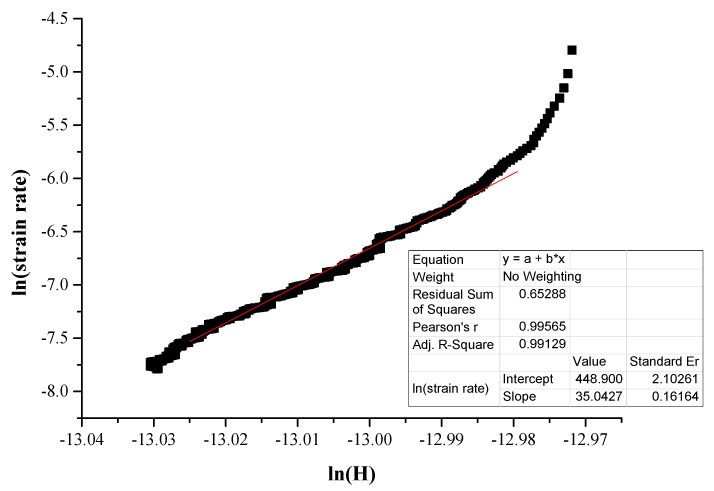
Linear fitting of steady creep area for actual stress exponent (*n*) slope calculation.

Figure 9 shows an example of the fitting curve along with the related parameters and Figure 10 and Figure 11 present examples of $\ln\left(\dot{\varepsilon }\right)$ versus ln⁡(H) curves.

Another important factor that can be calculated from m (strain rate sensitivity)—derived from Equation (11)—is the **critical volume for dislocation nucleation, *V_cr_*** [44,45].

(12) $$V_{cr}=\frac{kT}{\tau_{max}m}$$

where *k* is the Boltzman constant, *T* the temperature in Kelvin degrees and ***τ_max_*** the **maximum shear stress during the creep stage**.

According to Wang et al. [44]:

(13) $$\tau_{max}=\frac{H_{max}}{3\sqrt{3}}$$

and

(14) $$H_{max}=\frac{P_{max}}{24.5h_{0}^{2}}$$

***H_max_*** is calculated from Equation (14), where *P_max_* is the load when the indenter has reached the preset indentation depth (in this case 1000 nm) after which the creep stage commences, and *h*_0_ is close to this value. 

**Figure 12:**
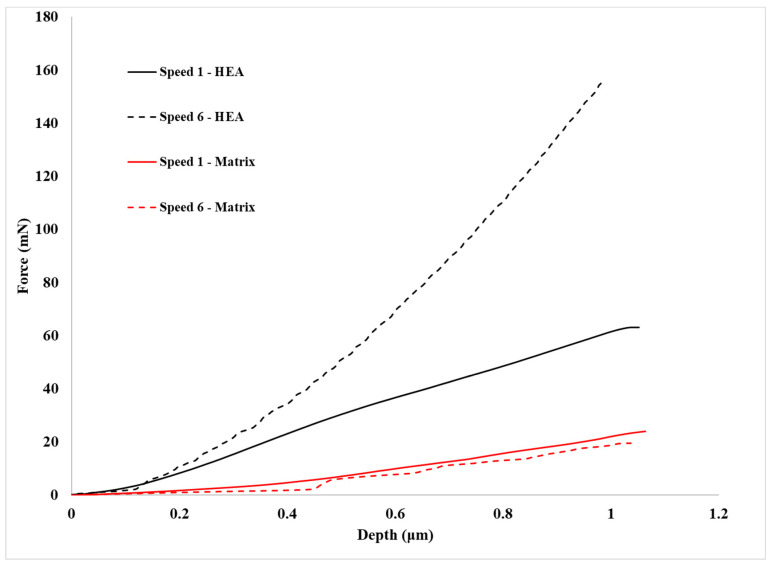
Loading stages for HEA, matrix and pure aluminum at two different speeds.

One of the most important issues before any attempt to assess the creep behaviour of both the matrix and the reinforcing phases is the examination of the loading stage prior to the actual creep stage. Figure 12 presents examples of the loading stage for the HEA reinforcement and the Al matrix. All loading curves are depicted as smooth without indications of load–depth fluctuations. In many research works [45,46,47], the presence of such fluctuations—also known as “serrations”—has been reported. Their presence is considered of significant importance as they are associated with the manifestation of micro-creep phenomena during loading and prior to the net/pure creep stage. As such, if present, they affect the material creep behaviour during the actual creep stage. In the present effort—and as Figure 12 depicts—such “serrations” do not exist. This absence shows that the dislocations, which are either primarily present in the microstructural phases from the manufacturing stage and/or generated during the loading stage, do not exert extensive mobility that could cause even limited/marginal creep deformation during the loading stage. It is also evident from Figure 12 that the different loading speeds do not seem affect the presence of absence of such load–depth fluctuations.

Another important detail that is provided by Figure 12 is the necessary load to reach the preset depth for both the Al matrix and the HEA reinforcement. Clearly, in the case of the Al matrix, this load is significantly lower than that of the HEA phase, a fact which is expected since Al is of soft and ductile nature compared to the HEA particles, which are very hard and brittle. Another important observation that must be mentioned is the effect the loading speed has on this load. It can be observed that in the case of the Al matrix, the necessary load is slightly lower at the lower loading speed. This is expected since, at lower speeds, (i) the available time for dislocation movement is prolonged and (ii) the possibility of extended dislocation entanglement that could restrain the ease of dislocation movement is reduced. Both these reasons enhance the ability of the indenter to reach the preset depth more easily, i.e., in terms of loads at lower loading speeds. In the case of the HEA reinforcing phase, however, it seems that the behaviour is reversed: the necessary preset depth load increases at the lower loading rates. This observation is in contradiction with previous work of Ananiadis et al. [19] where both the refractory HEA reinforcing particles and the Al matrix expressed the same tendency: lower loading speeds showed lower loads for the preset depth. The authors attribute this reversed reinforcement behaviour to the possible following reasons: 

In the previous work of Ananiadis et al. [19], the reinforcing phase was refractory MoTaNbVW particulates, solely of BCC crystal structure. In the present effort, the reinforcing phase is of hard AlMnCrFeNi HEA particulates. In another previous work, Ananiadis et al. [48] examined in depth the microstructural features of this bulk monolithic alloy and observed a complex in terms of morphology and phases alloy, consisting of a mixture of BCC B2 (BCC structured) and possibly FCC phases of very fine size. 

It is possible that this complexity of the involved multiple phases may play an important role in the loading behaviour. The presence of many phases leads to the establishment of multiple interfacial regions. It is possible that at high loading speeds, the involved dislocations are energetically enhanced to continue their movement and overpass effectively the obstacles these interfaces may pose. On the contrary, at lower loading speeds, the number of generated dislocations is reduced and/or their retarded movement may lead to their accumulation at the interfacial boundaries, inhibiting in such a way the ease of their further movement. The authors, nevertheless, do agree that further investigation is required to ascertain this behaviour. 

**Figure 13:**
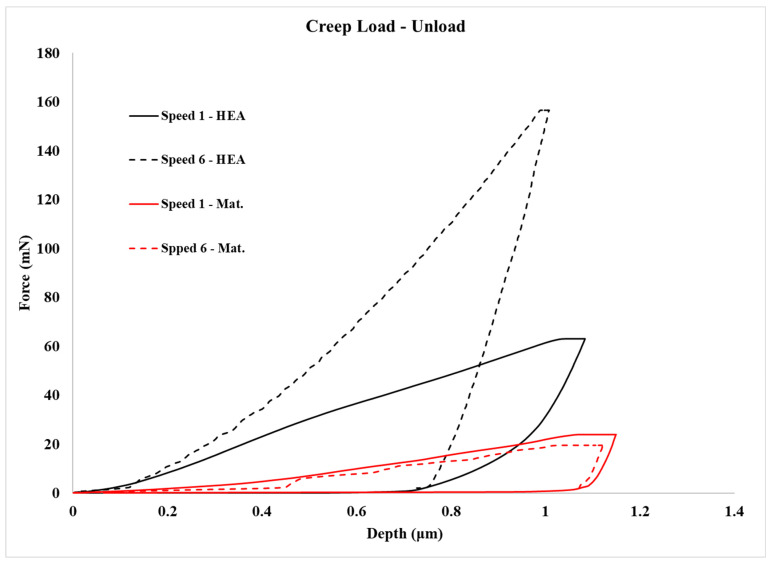
Creep indentation load–unload cycle curves for pure Al, matrix and HEA reinforcement.

Examples of overall load–creep–unload curves are presented in Figure 13. Clearly, the previously mentioned observations on the effect of loading speed on the loads to achieve the preset depth are depicted.

**Figure 14:**
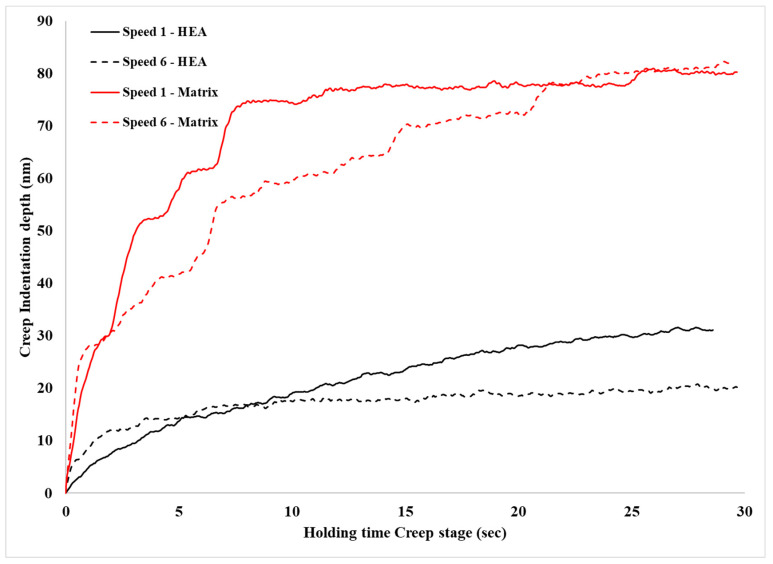
Actual depth of creep indentation as a function of holding time.

**Figure 15:**
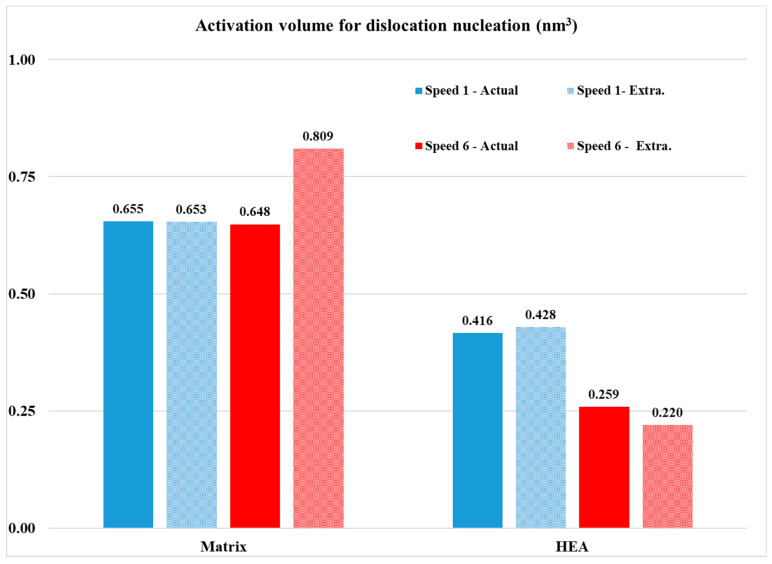
*V_cr_* comparison for the three areas of interest at two different creep speeds for actual and extrapolated values.

The necessary assessment of the creep behaviour data is summarized in Table 3. Figure 14 shows examples of *h_creep_*—time curves for the different systems examined in the present effort—and Figure 15 presents the *V_cr_* values of the different phases in this work. In the following paragraphs, various important points derived from Table 3 and Figure 7 and Figure 8 are discussed.

Concerning the actual creep depth, it can be seen from Table 3 that in the case of the reinforcing phase, the creep depth decreases at the lower loading speed, whereas in the case of the matrix phase, it seems that the loading speed does not have a significant effect on the creep depth. Additionally, it can also be observed that the creep depth of the matrix is considerably higher than that of the reinforcing HEA. This is expected due to the matrix’s soft and ductile character compared to the hard and stiff nature of the reinforcing HEA particles. This trend of the net creep depth is further verified by the values of the n exponent, presented in Table 3. The *n* exponent is directly related to the creep behaviour: the higher its value, the more difficult for the creep phenomena to take place [37,38,39,40,41,45]. Indeed, in the present work, the n exponent possesses values within the range of 30–35 (both actual and extrapolated) for the matrix phase with the higher creep depths and values of 58–86 (both actual and extrapolated) in the case of the HEA reinforcing phase that showed the lower creep indentation depths.

In order to further approach the creep response of the involved phases, the *τ_max_* and the *V_cr_* parameters should also be taken into consideration. Let us consider firstly the case of the reinforcing phase. It is shown that this phase has the lower creep depth. As shown in other experimental works [37,38,39,41,42,44,45,46,47], the indentation creep behaviour is associated with the number and the mobility of the involved dislocations. It was shown that, at the lower loading speed, the creep depth was reduced. This practically means that the number of the available mobile dislocations that could lead to creep deformation during the creep stage is restricted. This is most likely associated with the observations mentioned previously concerning reaching the necessary preset depth load value: it was depicted that at lower speeds the load was increased, a fact that was associated with the complex nature of the multiple phases being present in the microstructure of the HEA reinforcement. As in that case, it is possible that at the lower loading speeds, the generated dislocations are blocked at the multiple interfaces and their mobility is limited. As such, more dislocations have to be generated during the creep stage in order to accommodate the creep deformation. This generation is easier at the high loading speeds and more difficult at the lower speeds, as mirrored by the *τ_max_* values: For the lower loading speed, *τ_max_* is 1.045; whereas in the case of the higher speed, the value is 0.741. This increased *τ_max_* results, nevertheless, in a balanced and similar *V_cr_* value (the threshold volume required for the generation of dislocations through which the creep deformation will be expressed). 

In the case of the Al matrix on the other hand, even though the creep depth is higher, the *τ_max_* values are significantly lower than that of the HEA phase, and the *V_cr_* values are significantly higher. This could be considered as a contradicting observation, yet this is not the case. The high creep depth for the Al matrix means that an increased number of mobile dislocations are available to accommodate the creep deformation. The low *τ_max_* values achieved force a higher volume to be stimulated in order to generate dislocations during the creep stage, i.e., the *V_cr_* values are increased. The high creep depth values, nevertheless, cannot be only the result of the mobile dislocations generated during the actual creep stage but most likely are a combination of dislocations available at both the loading and the creep stage. Similar postulates have been expressed in other experimental works [37,38,39,41,42,44,45,46,47].

### 3.3. Sliding Wear Response

**Figure 16:**
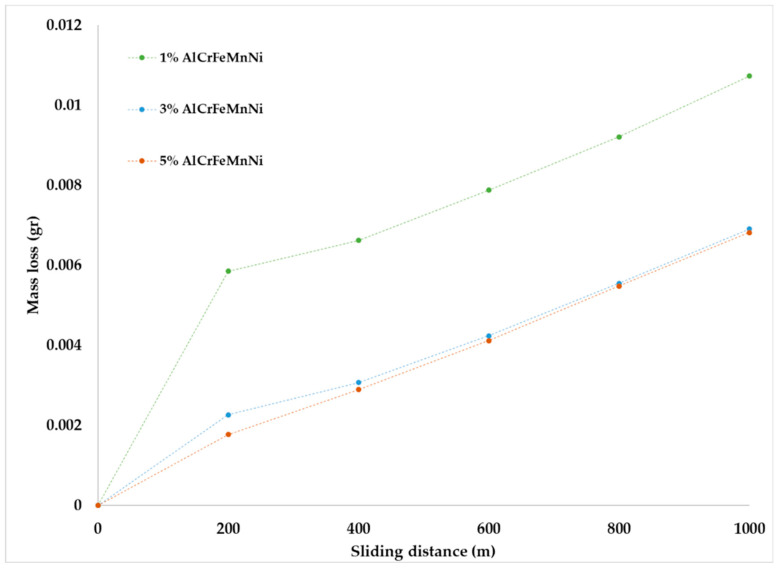
Mass loss versus sliding distance for each composite system.

**Figure 17:**
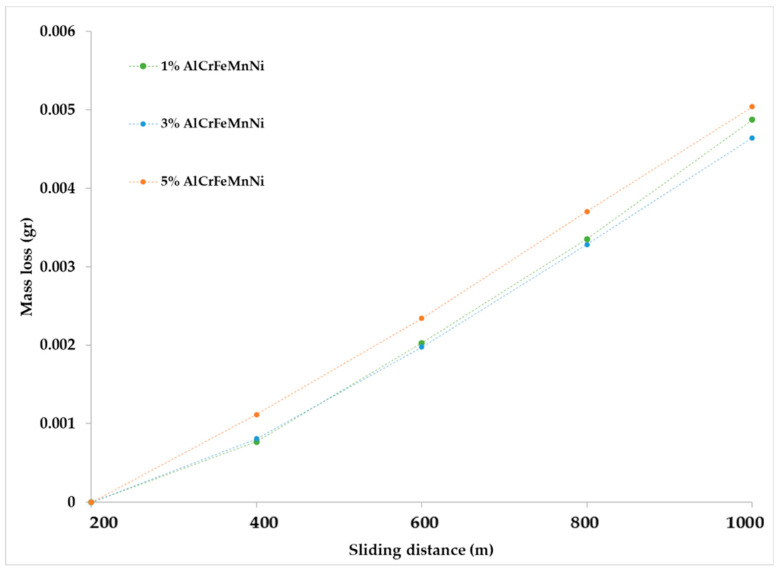
Mass loss versus sliding distance for each composition after the first 200 m.

**Figure 18:**
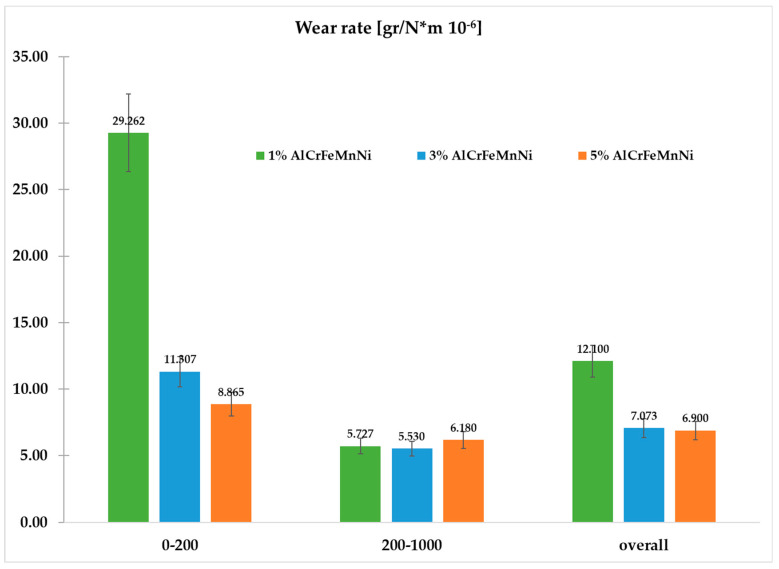
Wear rates for the initial 200 m, 200 to 1000 m and overall sliding distance of each composition.

The mass loss versus the sliding distance of the specimens is presented in Figure 16. It can be observed that during the first 200 m of sliding distance, all specimens show a relatively increased mass loss. This behaviour is due to the fact that in the initial stages of the sliding action, the primary asperities of the two counter surfaces collapse, leading to an extensive material removal. Similar observations are addressed in other experimental works [19,49,50]. After this initial stage and for the next 800 m, a stabilised contact between the two surfaces is established, a steady wear state is observed and the mass loss over distance shows a more linear and smoother behaviour (Figure 16). Other experimental works also present similar trends [19,49,50]. The analysis of the steady state behaviour (200–1000 m) is presented more clearly in Figure 17. For a more detailed reader’s undesrtanding, Figure 18 shows the overall wear behaviour for (i) the initial 200 m, (ii) the 200–800 m region and (iii) the overall (0–1000 m) sliding distance.

Despite the reinforcement addition, the overall wear rates show a non-usual approach as the 1% reinforced sample seems to have the highest, as it was observed in previous work for a different composite [19]. This is happening as, in the initial 200 m of the experiment, the mass loss of the 1% sample is extremely high. Samples with increased reinforcement exhibit lower wear rates and thus have better wear response [51,52,53,54,55,56].

**Figure 19:**
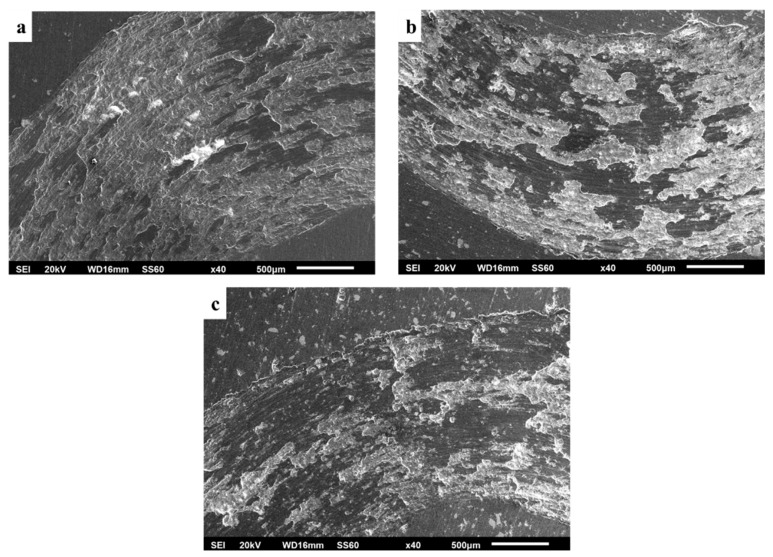
Panoramic view of worn surfaces for: (**a**) 1 vol.%, (**b**) 3 vol.% and (**c**) 5 vol.% specimens.

Figure 19a–c show a panoramic view of the wear tracks for the different systems examined in the present effort. Two different morphologies of the wear track can be observed in all cases: (i) dark shaded areas of smooth texture and non-intensive landscape and (ii) brighter rough areas of intensive landscape. The dark areas are associated with non-severely attacked regions of the surface, whereas the rough light areas are the results of an oxidation–delamination wear mode. It is worth mentioning that by increasing the reinforcement content, the areas of extensive mass removal (light areas) are considerably restricted. This is a strong indication of the beneficial role the reinforcing particles play in increasing the systems’ wear resistance. 

**Figure 20:**
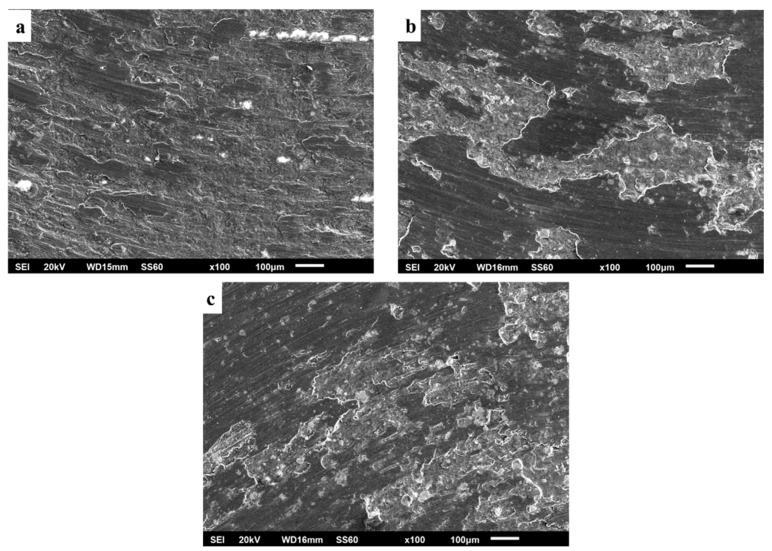
Wear tracks for each composition. (**a**) 1 vol. %, (**b**) 3 vol.% and (**c**) 5 vol.% at higher magnification.

**Figure 21:**
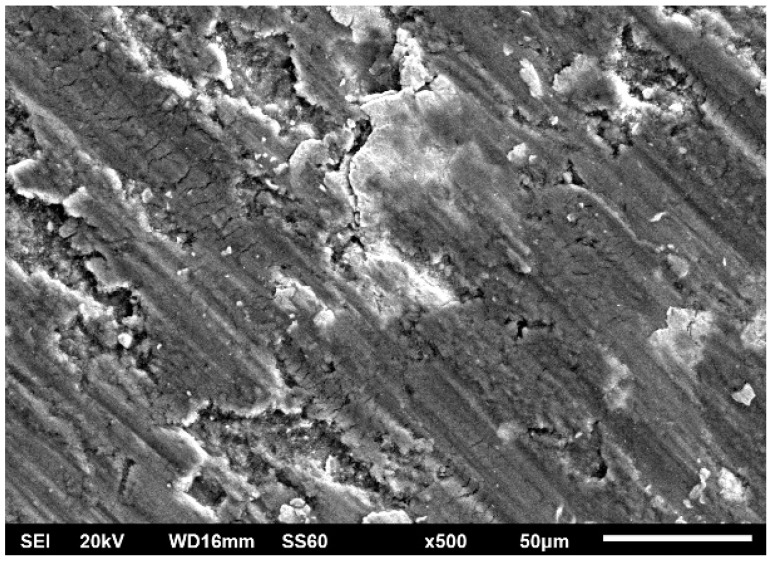
Surface cracks forming perpendicular to the sliding direction on the Al matrix (5 vol.%).

**Figure 22:**
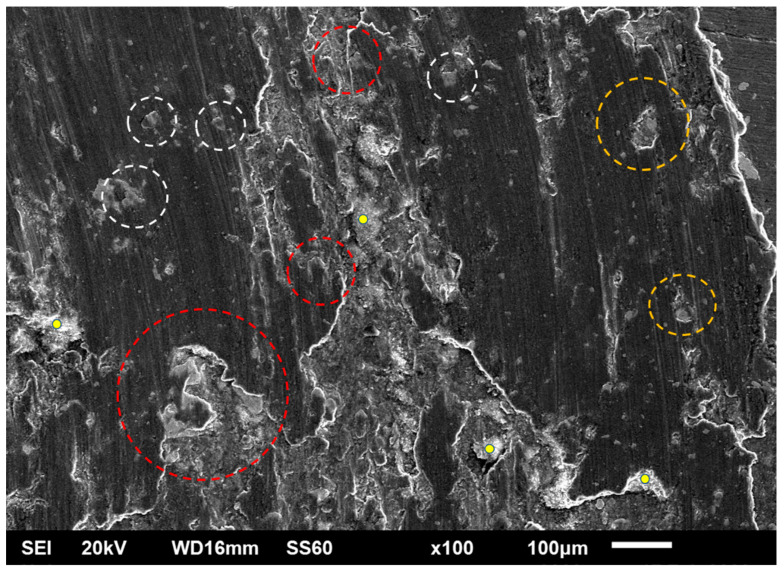
Areas of interest of the worn composite surfaces.

**Figure 23:**
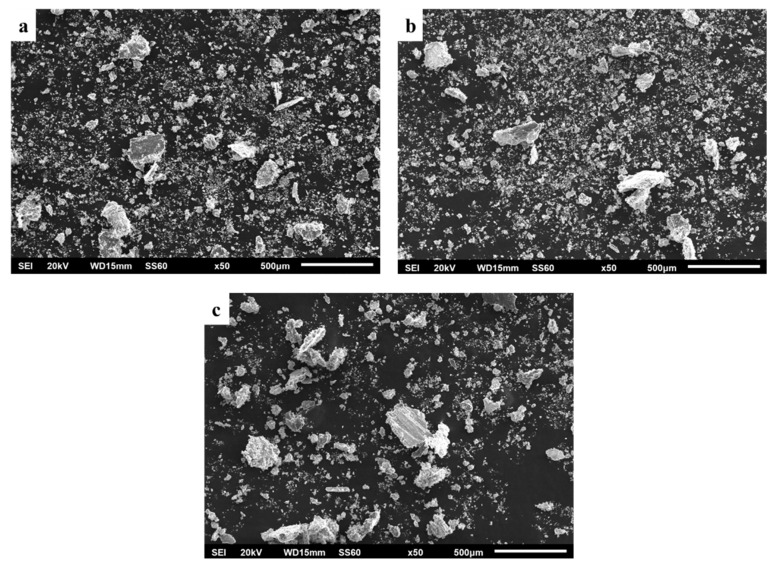
Wear debris from the surfaces for each composition. (**a**) 1 vol. %, (**b**) 3 vol.% and (**c**) 5 vol.%.

The worn surfaces presented in Figure 19 along with their appearance at higher magnification (Figure 20) depict that the material loss sequence is a combination of different wear mechanisms. As seen in Figure 20, the presence of scratches and grooves parallel to the sliding direction are an indication of an abrasive wear mode action. This wear mechanism is expressed and developed as hard particles consisting of both fragments of the reinforcing particles and debris generated by the oxidation of the surface abrade the soft matrix, leading to material removal [57]. On the other hand, the rough isolated lake-like bright areas on the dark surfaces are an indication of an oxidative wear mode. As the sliding action proceeds, the associated friction leads to temperature increase which in turn, as the experiment is conducted in an ambient environment, leads to the oxidation of the surface [58,59]. Surface oxides form a layer which fills and compacts surface crevices and expresses a strong lubrication action. Additionally, the presence of this oxide phase layer reduces the net metallic contact between the two counter-faces. Both these two reasons restrict the material loss and improve the wear resistance [60,61]. Nevertheless, this beneficial action is maintained if the integrity and the continuance of this protective layer is ensured. Any possible action that destroys the integrity of this layer (i.e., development of cracks and flaws) leads to extended removal of oxidised islands in the form of flakes, giving rise to the mechanism of delamination wear. Simultaneously, a new fresh metallic contact is re-established, and the cycle is repeated. The development of cracks/flaws that can destroy the integrity of the surface layer upon which, as the sliding proceeds and under the influence of the externally applied load, microcracks perpendicular to the sliding direction are formed, joined and propagated, leading eventually to material removal under the delamination mode [62], is considered to be the result of fatigue phenomena. The presence of such crack/flaw types are evident in Figure 21 and Figure 22. The morphology of flake-shaped debris associated with this mechanism is shown in Figure 23.

In summary, the three different material degradation mechanisms are depicted in Figure 21: (a) grooves parallel to the sliding direction indicating abrasive mode of material removal, (b) bandles of cracks indicating fatigue action associated with extensive oxidation at the lips of the cracks and (c) flake-like material removal traces indicating delamination wear.

Furthermore, the role of the reinforcing particles and the oxidation phenomena in the overall wear sequence are shown in Figure 22. Certain specific areas of interest are indicated: (i) yellow marks showing extensively oxidised areas that eventually detach by the surface, (ii) white circles indicating reinforcing particles where initially formed cracks vertical to the sliding direction are stopped from being propagated, (iii) yellow circles indicating areas of removed reinforcing particles (a possible mechanism of material removal can be as follows: a crack has approached the particle without transpassing it and, on the contrary, the crack was developed circumferentially to the particle-matrix interface, leading eventually to the removal of flake-like debris with this action, however, causing the delay of the material removal sequence) and (iv) large reinforcing particles located at the contour of the ridge of large removed flake debris. Their presence shows their beneficial action in retarding the degradation process.

The morphology of the produced debris is shown in Figure 23. As previously mentioned, large flake-like particles can be observed, supporting the assumption that delamination is one of the predominant mechanisms for these specimens. Finer particles are also present among the larger ones. These particles are most likely the result of a continuous grinding action on initial debris trapped between the two counter surfaces.

### 3.4. Corrosion Performance

**Figure 24:**
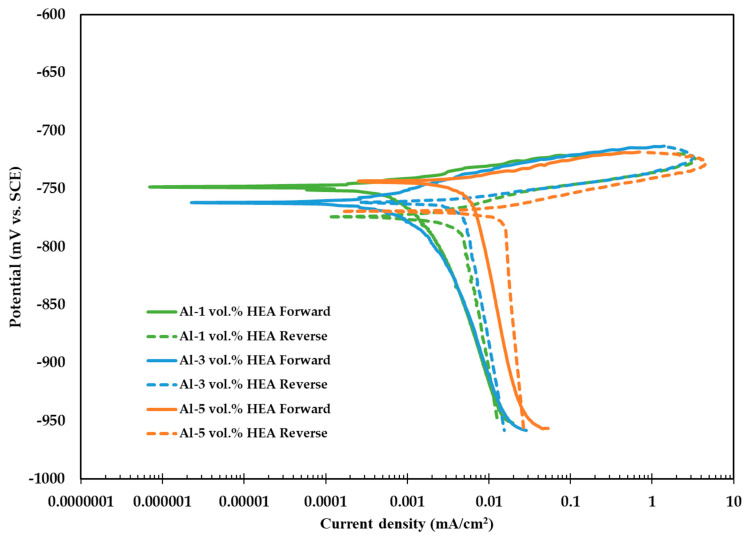
Potentiodynamic polarisation curves for Al-HEA composites (1–5 vol.%) in 3.5 wt.% NaCl solution (RT).

Figure 24 presents the potentiodynamic polarisation curves for the produced composites in 3.5 wt.% NaCl solution (room temperature). The curves are typical for Al composites and Al alloys in 3.5 wt.% NaCl solution [49,63,64]. In more detail, regardless of the reinforcement content, the composites exhibit a sustained flat gradient of the forward anodic polarisation curves, an indication of susceptibility to localised forms of corrosion in a saline environment. Another manifestation of this behaviour is that the reverse polarisation curves correspond to higher current density values as compared to the forward curves for the same potentials (formation of a negative hysteresis loop). The curves appear to be similar, indicating that the corrosion performance of the composite is not affected by the increasing volume of the reinforcement. It can thus be concluded that the corrosion performance of the material in the studied environment is mainly controlled by the corrosion of the matrix, in good agreement with similar works [29,34].

**Figure 25:**
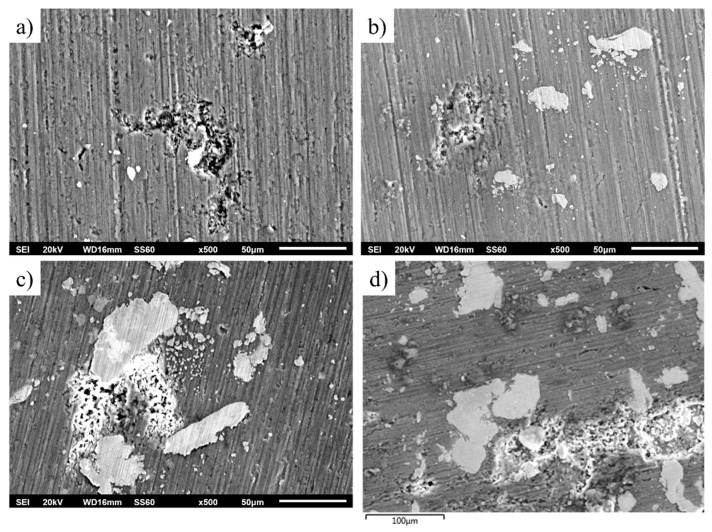
Corroded surfaces after potentiodynamic polarisation: (**a**) 1 vol.%, (**b**) 3 vol.%, (**c**,**d**) 5 vol.% HEA.

Analysis of the degraded surface after polarisation (Figure 25) indicates that the corrosion mostly occurred at the Al matrix, at the interface between particulates and the Al matrix. This is the outcome of galvanic corrosion and selective dissolution of the Al matrix. This is anticipated since the particulates contain elements nobler than the matrix such as Fe, Cr, Ni, and Mn. It is likely that the presence of Al in the particulates decreased the intensity of the galvanic effect. Additional galvanic couples were possibly created, such as between areas richer in the noble particulates and areas with a smaller volume of AlCrFeMnNi particulates. HEA particulates may enhance the corrosion performance by enriching the surface film with corrosion-resistant elements such as Cr or Ni. On the other hand, particulates introduce interphases on the surface film that are favourable sites for pit corrosion. Nonetheless, the surfaces exhibit a similar degree of degradation, another sign of the similar corrosion performance and similar degradation mechanisms of the composites.

## 4. Conclusions

Composites containing third transition metal-based HEA were fabricated successfully, and the mechanical and corrosive properties were evaluated.

The microstructure appeared to be homogenous, without any significant defects (i.e., microporosity).

No interfacial reaction products were observed between the matrix and the particles, indicating no diffusion or intermetallic phase formation.

Increase of the reinforcement phase led to an increase in the overall hardness of the fabricated composites.

Nanoindentation-based mechanical properties and creep response were associated with the nature of the Al matrix and the HEA reinforcing phase.

Wear behaviour is enhanced with increasing volume fraction of HEA particles.

Regardless of the reinforcement content, the composites exhibited similar corrosion performance. The corrosion behaviour was mainly controlled by the corrosion of the Al matrix.

## Figures and Tables

**Table 1:** Nanoindentation-based fundamental mechanical properties (loading speed 1).

Speed 1 (13.324 mN/s)			
	Hardness (HV)	*E_it_* (GPa)	*n_it_* (%)
**Matrix**	53.33 ± 3.74	93.13 ± 10.6	6.3 ± 4.74
**HEA**	735.8 ± 124	124.4 ± 39.5	26.03 ± 7.57

**Table 2:** Nanoindentation-based fundamental mechanical properties (loading speed 2).

Speed 6 (2.2207 mN/s)			
	Hardness (HV)	*E_it_* (GPa)	*n_it_* (%)
**Matrix**	44.54 ± 14.25	87.3 ± 13.3	4.72 ± 2.44
**HEA**	811.5 ± 190.8	157.56 ± 15.7	33.03 ± 6.96

**Table 3:** Creep response assessment data.

Phase	Loading Speed (mN/s)	*h_creep_* (nm)	*n* Actual	*n* Extrapolated	*m* Actual	*m* Extrapolated	*H_max_* (GPa)	*τ_max_* (GPa)	*V_cr_* Actual (nm^3^)	*V_cr_* Extrapolated (nm^3^)
HEA	13.32	31.6	58.164	65.151	0.024	0.017	3.709	0.714	0.275	0.350
	2.22	18.9	85.7	72.112	0.012	0.015	5.43	1.045	0.259	0.22
Matrix	13.32	80.9	29.194	29.194	0.036	0.036	0.679	0.131	0.653	0.655
	2.22	81.5	28.165	35.057	0.039	0.033	0.642	0.124	0.648	0.809
*n* actual, *m* actual, *V_cr_* actual: Parameter values calculated based on the actual *h creep* depth as a function of time received during testing										
*n* extra, *m* extra, *V_cr_* extra: Parameter values calculated based on the extrapolated values of the *h creep* as a function of time after fitting by Equation (4)										

## Data Availability

Not applicable.
